# Endothelial glycocalyx in traumatic brain injury associated coagulopathy: potential mechanisms and impact

**DOI:** 10.1186/s12974-021-02192-1

**Published:** 2021-06-14

**Authors:** Zhimin Zou, Li Li, Nadine Schäfer, Qiaobing Huang, Marc Maegele, Zhengtao Gu

**Affiliations:** 1 Academy of Orthopedics, Guangdong Province, Guangdong Provincial Key Laboratory of Bone and Joint Degenerative Diseases, The Third Affiliated Hospital of Southern Medical University, Guangzhou, Guangdong 515630 China; 2 Department of Treatment Center for Traumatic Injuries, The Third Affiliated Hospital of Southern Medical University, Guangzhou, Guangdong 515630 China; 3 Guangdong Provincial Key Lab of Shock and Microcirculation, Department of Pathophysiology, School of Basic Medical Sciences, Southern Medical University, Guangzhou, 510515 China; 4 Institute for Research in Operative Medicine (IFOM), University Witten/Herdecke (UW/H), Campus Cologne-Merheim, Ostmerheimerstr. 200, D-51109 Köln, Germany; 5 Department for Trauma and Orthopedic Surgery, Cologne-Merheim Medical Center (CMMC), University Witten/Herdecke (UW/H), Campus Cologne-Merheim, Ostmerheimerstr. 200, D-51109 Köln, Germany

**Keywords:** Traumatic brain injury, Coagulopathy, Endothelial glycocalyx

## Abstract

Traumatic brain injury (TBI) remains one of the leading causes of death and disability worldwide; more than 10 million people are hospitalized for TBI every year around the globe. While the primary injury remains unavoidable and not accessible to treatment, the secondary injury which includes oxidative stress, inflammation, excitotoxicity, but also complicating coagulation abnormalities, is potentially avoidable and profoundly affects the therapeutic process and prognosis of TBI patients. The endothelial glycocalyx, the first line of defense against endothelial injury, plays a vital role in maintaining the delicate balance between blood coagulation and anticoagulation. However, this component is highly vulnerable to damage and also difficult to examine. Recent advances in analytical techniques have enabled biochemical, visual, and computational investigation of this vascular component. In this review, we summarize the current knowledge on (i) structure and function of the endothelial glycocalyx, (ii) its potential role in the development of TBI associated coagulopathy, and (iii) the options available at present for detecting and protecting the endothelial glycocalyx.

## Introduction

Traumatic brain injury (TBI) is still one of the leading causes of death and disability worldwide with more than 10 million people hospitalized every year [1]. TBI refers to damage to the brain caused by any external mechanical force which may cause injury, for example rapid acceleration or deceleration, waves of shock, extrusion, impact, or penetration of projectiles and other objects [2]. Despite its high prevalence and cost for both, individuals and society, fundamental and underlying pathophysiology remain yet poorly understood and effective therapeutic options are still limited. Alterations of the coagulative system and disturbed coagulation function are common findings in patients with TBI; two out of three patients with severe TBI display abnormalities on conventional coagulation tests on admission which translate into poorer outcomes [3, 4]. TBI-associated coagulopathy may refer to both hypocoagulopathy with prolonged bleeding and hemorrhagic progression and hypercoagulopathy with an increased prothrombotic tendency, both of which can occur—either simultaneously or sequentially—after impact [5, 6].

Various mechanisms potentially linked to hemostatic disturbance after TBI have been studied, among which endothelial cell activation and glycocalyx shedding are currently given priority for further exploration [6, 7]. Poorer outcomes after isolated blunt TBI were associated with rapid increase in circulating biomarkers indicative for both endotheliopathy and coagulopathy according to the magnitude of injury. These profiles confirmed hemostatic failure, endothelial damage including damage to the inner endothelial layer (glycocalyx) injury, inflammation, and hyperfibrinolysis within the first 24 h after TBI [8]. Shedding of the glycocalyx seems capable to trigger systemic thrombin generation, activation of protein C, and hyperfibrinolysis in addition to potentially direct anticoagulative effects from endogenous heparinization. However, the shed glycocalyx may also increase circulating concentrations of damage-associated molecular patterns (DAMPs) including hyaluronan fragments and heparin sulfate. These DAMPs may then contribute to a hypercoagulative state through the production of inflammatory mediators and subsequent thrombin generation. Indeed, previous studies have shown glycocalyx disruption increases thrombin production [9].

In order to better understand the differences in occurrence and phenotype of TBI associated coagulopathy, it is necessary to further explore the structure and function of the glycocalyx, and to gain more in-depth knowledge about the potential mechanisms behind glycocalyx damage and down-stream consequences in the context TBI. In this review, we summarize the current knowledge on (i) structure and function of the endothelial glycocalyx, (ii) its potential role in the development of TBI associated coagulopathy, and (iii) the options available at present for detecting and protecting the endothelial glycocalyx.

## Structure of the glycocalyx

The endothelial glycocalyx (EG) represents a layer of negatively charged, brush-like polysaccharide-protein complex structures on the surface of endothelial cells [10]. This layer of membrane-bound, carbohydrate-rich molecules covers the luminal surface of the endothelium along the entire vascular tree, mostly comprising glycoproteins and proteoglycans. Together with the underlying actin-rich endothelial cortex, 50 to 150 nm underneath the plasma membrane, EG is recognized as a vasoprotective nanobarrier and responsive hub; it presents highly dynamic and can adapt its nanomechanical properties (i.e., stiffness and height) in response to environmental changes [11]. The constant change between soft and stiff endothelial surfaces is imperative for proper functioning of the endothelium. Recently, EG has been defined as a critical component of the expanded neurovascular unit that influences the structure of the blood-brain barrier and plays various physiological functions, including an important role in maintaining normal neuronal homeostasis, which is related to maintaining the integrity of the blood vessel wall [12, 13]. However, the tiny structure and more nuanced functions of EG have been greatly ignored for a long time.

**Fig. 1:**
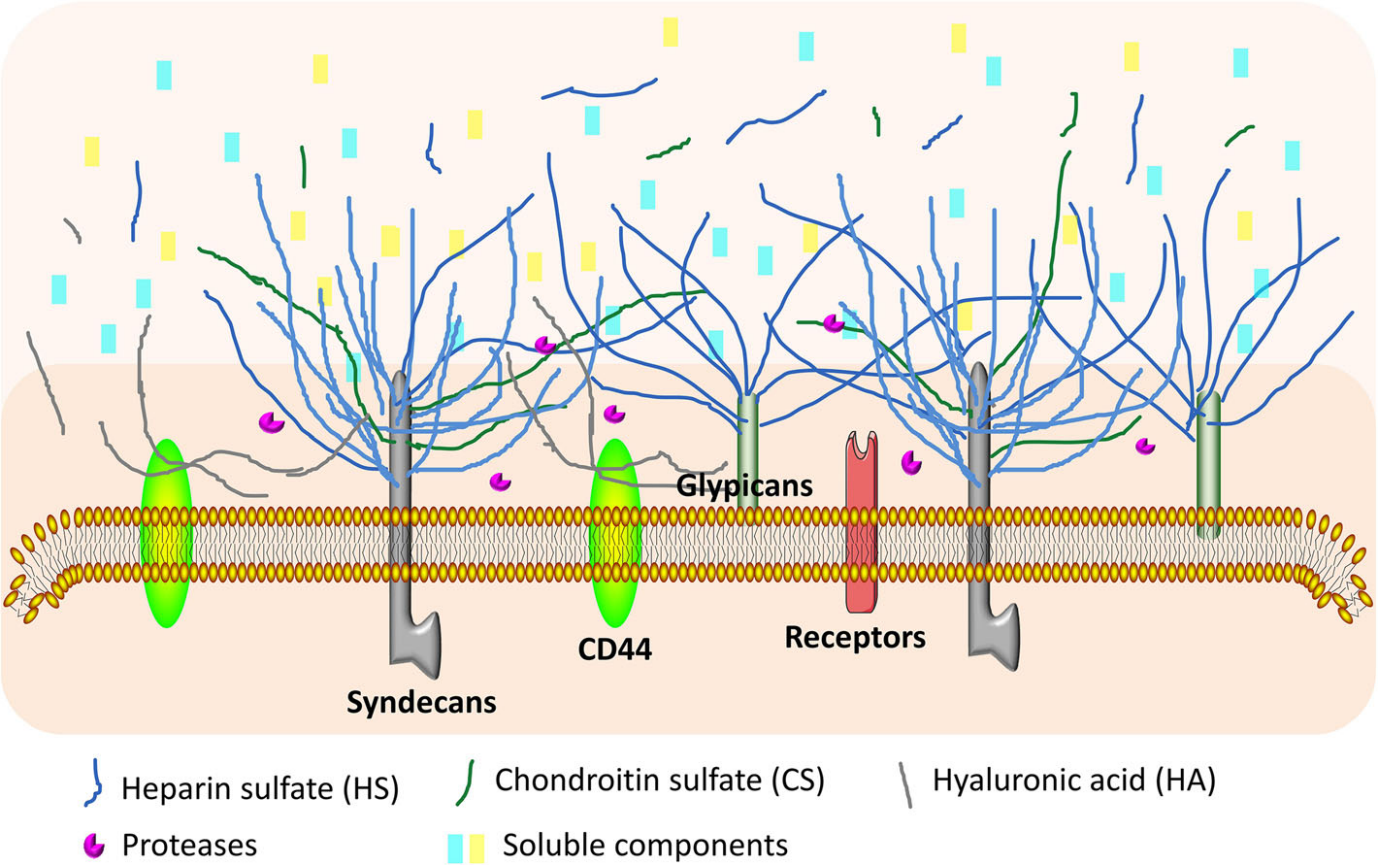
Schematic representation of the endothelial glycocalyx. Bound to the endothelial membrane are proteoglycans, with glycosaminoglycan side-chains (GAG-chain) and glycoproteins. Incorporated in and on top of this grid are plasma and endothelium-derived soluble components and various proteins. Together, these components form the endothelial glycocalyx that functions as a barrier between blood plasma and the endothelium and exerts various roles in plasma and vessel wall homeostasis

The concept of the thin endothelial layer was first proposed in 1940 [14], but it was not until 1966 that Luft and co-workers used an electron microscope to observe the glycocalyx structure of about 20 nm EG in rat intestinal mucosal blood vessels through ruthenium red staining [15]. With advanced observation technologies and research methods, the structure and function of glycocalyx have been gradually understood [16–18]. To date, it is believed that glycocalyx composition may vary according to tissue and cell type. The components of EG depend on plasma composition and local hemodynamic conditions and are composed of two layers [19, 20], e.g., (i) an inner-dense matrix layer: A relatively thin area of the glycocalyx (50–100 nm) composed primarily of “side chain”-glycosaminoglycans (GAGs) and “skeleton”-proteoglycans (PGs) directly bound to the plasma membrane and strictly glycocalyx, and (ii) an outer less-dense layer: a thicker area (0.5 μm) attached to the glycocalyx, which is a complex three-dimensional structure composed of soluble plasma components showing dynamic equilibrium (Fig. 1). The PGs skeleton bound to cell membrane and the GAG chain linked to the skeleton form a grid structure; incorporated in and on top of this grid are plasma and endothelium-derived soluble components and various proteins. Together, these components form the endothelial glycocalyx that functions as a barrier between blood plasma and the endothelium and exerts various roles in plasma and vessel wall homeostasis.

### “Side chain”

Glycosaminoglycan (GAG) is considered as the most abundant component of the glycocalyx, mainly including hyaluronic acid (HA), heparin sulfate (HS), and chondroitin sulfate (CS). In the vascular system, HS accounts for 50–90% of the entire GAG. Quantitatively, HS and CS are usually present in a ratio of 4:1, which is altered under various stimuli, e.g., endothelial cell activation or injury. Except for HS, other GAGs are sulfated structures. Because GAGs contain a number of specific binding sites for plasma proteins, the enzymatic chemical modification and different sulfation patterns of sugar molecules can endow proteoglycans with unique physiological functions. These modification patterns may change over time under different pathophysiological stimuli, affecting vascular permeability and the binding activity of specific proteins.

### “Skeleton”

Proteoglycan is usually considered as the most important “skeleton” molecule of the glycocalyx. It covalently binds to one or more GAGs through the core protein to form a kind of glycoconjugate, e.g., heparan sulfate proteoglycan (HSPG) [21, 22]. The type and number of glycosaminoglycan chains attached to different core proteins and whether they are bound to the cell membrane are not the same. Proteoglycan core proteins can be divided into membrane proteoglycans and extracellular matrix proteoglycans according to their localization in the cell microenvironment [22]. The former is represented by the syndecan family of transmembrane multi ligand proteoglycans connected with the cytoskeleton and the glypican family anchored on the endothelial cell membrane; the latter represents various secretory matrix proteoglycans. The syndecan family members expressed by endothelial cells (ECs) are Syndecan-1, -2, and -4. Its structure is divided into three segments: (i) the intracellular segment, (ii) the transmembrane domain, and (iii) the extracellular segment [23].

The extracellular matrix proteoglycans have three GAG attachment sites, wherein the endothelial cell syndecan-1 can bind to HS and CS. Glypican-1 is the only member of the glypican family that is expressed on endothelial cells [22]. It is different from syndecan-1 in that it only has an HS chain and no CS chain. HA is a non-sulfated secreted GAG, which is not linked to the PG core protein and binds to the receptor CD44. In addition, some glycoproteins are also considered as “skeletal structures” that connect the glycocalyx to the endothelial cell membrane, including endothelial cell adhesion molecules (E-selectin, P-selectin) and some glycoproteins with functions for coagulation and fibrinolysis. Their expression levels undergo significant alterations under stimulation [24].

### “Dynamic soluble components”

Many soluble components, such as hyaluronic acid, albumin, thrombomodulin, extracellular superoxide dismutase, and antithrombin III, are embedded in the sieve structure formed by proteoglycans and glycoproteins [25, 26]. These soluble proteins and proteoglycans not only combine with each other to form a network to maintain the stability of the glycocalyx structure but also have significance in maintaining vascular permeability, anti-oxidation, and anti-coagulatio n[27, 28].

## Function of glycocalyx

### Natural barrier function

The glycocalyx is located at the interface between the blood flow and endothelial cells. Due to its particular spatial position and complex structure, it forms a natural mechanical barrier for endothelial cells, which can (i) reduce the risk for direct damage to endothelial cells by potentially harmful components in the blood stream, (ii) prevent receptor-mediated damage signal activation, and (iii) interaction between blood cells and the vascular wall. Under physiological conditions, the EG is much thicker than cell adhesion molecules in diameter, e.g., 0.2–0.5 μm in capillaries, 2–3 μm in arterioles, and 4.5 μm in carotid arteries [29, 30]. However, adhesion molecules such as P-selectin are only about 38 nm above the surface of endothelial cells. The GAGs and soluble component of glycocalyx seem to be capable to shield adhesion molecules and prevent the adhesion of erythrocytes, leukocytes, and platelets to endothelial cells [29, 31]. Glycocalyx can also serve as storage for functional proteins, such as galectins, which can be released and transferred to cells to perform their respective functions under stimulating conditions. In addition, there exists a buffer space between the glycocalyx and the blood vessel, and its colloid osmotic pressure is lower than the osmotic pressure of plasma and tissue gaps thereby acting as vascular barrier and preventing blood vessel leakage [32, 33].

### Regulation of vascular permeability

The permeability of the glycocalyx to substances mainly depends on two aspects: (i) molecular weight and (ii) charge. Glycocalyx contains a large number of negatively charged residues such as sialic acid, sulfated polysaccharide, and uronic acid, and its complex network structure can act as a molecular sieve, which prevents negatively charged macromolecules, white blood cells, red blood cells, and platelets from passing through the blood vessel wall [32]. Vink and Duling [34] used a variety of plasma tracers to probe the barrier properties of the glycocalyx using combined fluorescence and brightfield intravital microscopy and found no permeation of the endothelial surface layer for either neutral or anionic dextrans ≥ 70 kDa, but for neutral Dextran 40 (40 kDa) and neutral free dye (rhodamine, 0.4 kDa) equilibrated with the endothelial surface layer within 1 min. In contrast, small anionic tracers of similar size (0.4–40 kDa) permeated the endothelial surface layer relatively slowly with half-times (τ50) between 11 and 60 min, depending on tracer size. Furthermore, fibrinogen (340 kDa) and albumin (67 kDa) moved slowly into the endothelial surface layer at comparable rates. The glycocalyx is semi-permeable to albumin which has amphoteric characteristics and can be tightly combined with the glycocalyx, then reduces its permeability [32]. These findings suggest that multiple factors may influence the permeability of the barrier, including molecular size, weight, charge, and structure.

In addition to the glycocalyx itself, the adsorbed plasma components may also play a role for microvascular permeability. It has been shown that a variety of soluble plasma components and selected synthetic substances can reduce the permeability of endothelial cells, including albumin, orosomucoid (ORM), hydroxyethyl starch (hetastarch, HES), and cationized ferritin among others [35]. Haraldsson and Rippe [36] have assessed the perfusion properties of rat skeletal muscle microvessels and found that when serum was added to the perfusion solution containing albumin, even if the serum concentration was only 10%, the permeability of the perfusion solution was reduced fourfold. In another set of studies in which orosomucoid was added to the albumin solution, it was found that compared with albumin alone, the permeability was reduced. However, the degree of permeability change was different from that when serum was added, indicating that not a single component can maintain low permeability, but most of them are negatively charged substances and glycoproteins with a molecular weight > 10 kDa.

### Effect on hemodynamics

Within the circulatory system, local blood viscosity and hematocrit, at least to some extent, may also seem to be regulated by glycocalyx. It has been shown that in capillaries with a diameter of 5 μm, provided a 0.5-μm glycocalyx layer, the flow resistance may be increased threefold and the hematocrit can be reduced by at least 30% [37]. Compared with a smooth glass tube, even at the same red blood cell velocity, the presence of glycocalyx will reduce the flow volume of plasma [37]. Based upon hemodynamics and hematocrit within the physiological microvascular network, and considering the elasticity of the glycocalyx, Pries and co-workers [38] have added the actual glycocalyx thickness to the ideal model of mesenteric microvascular and found that the apparent viscosity of blood increased twofold, which laid the foundation for establishing a rheological model in vitro to be closer to physiological conditions in vivo.

### Mechanoreceptor properties

The endothelial glycocalyx may act as a mechanoreceptor on endothelial cells lining every blood vessel wall which are consistently exposed to the mechanical forces generated by the blood flow [39]. An increased shear stress may increase nitric oxide (NO) production, which, in turn, leads to vessel dilation and reduced adherence of leukocytes and platelets [30]. Higher shear stress is also known to increase albumin uptake and alter glycocalyx properties, e.g., increased thickness [40]. When the glycocalyx is intact, shear stress can be transmitted to the actin cytoskeleton or directly to the cell membrane through the core protein, thereby mediating cell signal transduction [41, 42]. Damage to the glycocalyx impairs these mechanisms and perturbs the endothelial response to mechanical stress. Besides physical stress, the binding of ligands and enzymes to the glycocalyx may also induce signal transduction and enzymatic modification [43, 44]. The activity of growth factors, such as fibroblast growth factor (FGF) and epithelial growth factor, depend on the interactions with the glycocalyx. EG repair would be promoted by activation of endothelial growth factor signaling by highly-sulfated HS fragments released into the circulation during EG degradation [44]. The mechanism of glycocalyx participation in mechanical force transmission can be linked to both the decentralized and centralized mechanisms of mechanotransduction put forth by Davies and co-workers [45]. Syndecans that contain both HS and CS have an established association with the cytoskeleton and can decentralize the signal through multiple site distribution within the cell, e.g., nucleus, organelles, focal adhesions, and intercellular junctions [42]. These findings suggest that the glycocalyx may act as a receptor for physical and chemical signals, thereby inducing the physiological responses of the vascular endothelium.

### Anticoagulantive function

The maintenance of its antithrombotic property is a key feature of the glycocalyx. The intact endothelium exerts range of anticoagulant properties, including production and release of NO, prostacyclin, and tissue factor pathway inhibitor (TFPI) [46]. Endothelial cells may also secrete heparan sulfate, which augments the anticoagulant action of antithrombin. Reportedly, circulating antithrombin in plasma may bind to heparan sulfate located on the luminal surface and in the basal membrane of the endothelium [27]. Thrombomodulin is another endothelium-bound protein with anticoagulant and anti-inflammatory functions that is triggered in response to systemic procoagulant stimuli [47]. Together with these critical anticoagulative functions, the glycocalyx plays an essential role in maintaining blood flow in the microcirculation. After TBI, the disruption of the glycocalyx loses its protective effects from the endothelial surface, allowing leukocyte and platelet adhesion and microthrombi formation [48, 49]. Anticoagulative substances, for example antithrombin III (AT III), heparin cofactor II and thrombomodulin, are lost after glycocalyx destruction, resulting in an imbalance between pro- and anticoagulation [50]. Furthermore, when the glycocalyx is shed, the anticoagulant activity of heparin and chondroitin sulfate in the side chain structure is retained, which may release into the blood circulation and induce autoheparinization [51]. Typically, the excessive activation of coagulation and pathological hyperfibrinolysis lead to disseminated intravascular coagulation (DIC), and the disruption of the glycocalyx contributes to this state. Gonzalez and co-workers have linked syndecan-1 levels not only to the severity of TBI but also to the development of coagulopathy [49].

## Evaluating the endothelial glycocalyx

### Experimental studies

Direct visualization and corresponding techniques are crucial for the assessment and characterization of the glycocalyx. The glycocalyx can be labeled through specific markers that attach to one or more of its components, making them fluorescent or detectable. The first imaging studies of the glycocalyx based upon transmission electron microscopy (TEM) date back to the year 1966 and have used ruthenium red; since then, many other attempts were made to visualize the glycocalyx using TEM and improved staining protocol [52, 53]. To date, electron microscopy is still the gold standard for visualization, even though many other research techniques have been applied for the detection of the glycocalyx. An alternative method, e.g., intravital microscopy, enables the observation of capillary blood flow in living animals. Brightfield and fluorescent microscopy can demonstrate the virtual diameter of capillaries in vivo, which are comparable to anatomical diameters [54]. The functional diameter of capillaries can also be estimated by measuring the diameter of deformed erythrocytes flowing through capillaries. The thickness of the EG can be calculated from the difference between functional diameters and anatomical diameters of capillaries [55]. In addition, the double tracer dilution method by using two tracers respectively label erythrocytes and endothelial cells to quantify the virtual volume of EG within the circulatory system [56]. Although the intravital microscopy and double tracer dilution method do not damage the integrity of the glycocalyx, they cannot be used to study cells in vitro. This can be performed by the application and development of flow cytometry and laser confocal fluorescence microscopy. However, there are still many limitations that need to be further surmounted.

While the techniques mentioned above may be beneficial for quantitative and qualitative assessment of EG, they do not completely represent the EG´s functional aspect. At present, controversy exists in the literature concerning the thickness of the glycocalyx, which can be interfered with by many factors, such as the different techniques applied, EG observed in vivo or in vitro, and even dimensions vary for different vascular beds, different flow rates, different organs, and in different species [52]. Hall and co-workers [53] have shown that the brain capillary glycocalyx thickness may differ in different sections of brain capillaries after TBI. Moreover, the in vitro model of cultured EG could not express glycocalyx with the same thickness as the glycocalyx found in vitro. Chappell and co-workers [57], by using electron microscopy of fixed umbilical vein endothelial cells, showed that the average thickness of the glycocalyx was 878 nm, while cultured human umbilical vein endothelial cells (HUVECs) showed that the thickness of glycocalyx was only 29 to 118 nm. Earlier studies have also suggested a disparity in vascular permeability between cultured ECs and the in situ microvascular wall, as evidenced by a twofold increase in the flux of albumin across cultured cells compared to that in capillaries [56], which may be attributed, at least in part, to differences in the structure of the overlying glycocalyx. Obviously, in vitro models failed to replicate the in vivo structure of the glycocalyx; whether the in vitro model can reflect the function of the glycocalyx in vivo is also worth pondering.

### Clinical studies

Rapid and “point-of-care” (POC) assessment of the EG may be of high clinical value in the care and outcome for TBI patients and potentially would open new therapeutic windows of opportunity [58]. However, the direct visualization of the glycocalyx in humans remains extremely difficult in vivo, mostly due to its fragility. At present, indirect methods are predominantly used to evaluate glycocalyx in humans.

A variety of noninvasive microscopic camera techniques have been used to visualize sublingual microcirculation in patients and to assess the glycocalyx in human sublingual microvasculature by recording perfused boundary regions (PBR) which refer to the penetration extent of flowing red blood cells (RBCs) into the EG [59–61]. Examples of these techniques include orthogonal polarization spectral (OPS), sidestream dark field (SDF), or incident dark field (IDF) imaging, There have also been put forward a range of other comprehensive approaches to bedside for in vivo sublingual microcirculation image capture and analysis techniques in the human clinical setting, but the microcirculatory imaging systems and the availability of automated software for the analysis of glycocalyx thickness still need further improvement for facilitating glycocalyx studies in humans. In addition, as the dynamic equilibrium with the glycocalyx components in plasma, humoral markers have been assessed for their ability to estimate glycocalyx shedding in vivo; these markers include syndecan-1 (also known as CD138), HA, HS, and CS [58]. However, none of these markers have been found to be endothelial-specific, thereby limiting their use in clinical research. In addition, the comparability of results is complicated by the lack of reliable enzyme-linked immunosorbent assay kits, and interpreting the results of such assays remains difficult because of the rapid, or yet unknown, turnover rate of these markers and the impact of hepatic uptake in their metabolism. After the tight link between the endothelial and RBCs glycocalyx was demonstrated, it has been suggested that routine clinical evaluation of erythrocyte glycocalyx might be a suitable method for evaluating the EG [61].

## Damage and protection of the glycocalyx in TBI

Damage to the glycocalyx can be found in the pathological sequelae of many acute and chronic diseases, such as trauma [62], sepsis [50], diabetes [63], atherosclerosis [64], nephropathy [65], and tumors [66]. It has been shown that the glycocalyx layer becomes thin, followed by destruction and shedding of its components. Traumatic brain injury unifies both the common characteristics of trauma and its particularities. Blood brain barrier (BBB) disruption is a major pathophysiological feature of TBI and is associated with neuroinflammatory events contributing to brain edema and cell death [67]. Both astrocytes and microglia can rapidly respond to injury by increasing the production of multiple factors that may have a significant effect on BBB function [68]. EG influences the structure of the BBB, which is related to maintaining the integrity of the blood vessel wall [12]. However, the primary injury of TBI leads to damaged endothelial cells and EG shedding, thus leading to a loss of barrier integrity and subsequent elevated permeability [49]. BBB breakdown not only triggers leukocyte recruitment and the migration of inflammatory cells activating astrocytes, but it also causes the release of pro-inflammatory cytokines, cytotoxic proteases, and reactive oxygen species (ROS) to activate microglia, affecting neuronal activit y[67]. Moreover, local inflammation occurs, expanding the site of injury, exacerbating the damage neutrophils into the damaged area of the CNS and inflammatory mediators are released into the systemic circulation. This set of events promotes the onset of a vicious cycle, which helps further damage to the BBB and even systemic secondary injury [67, 69].

**Fig. 2:**
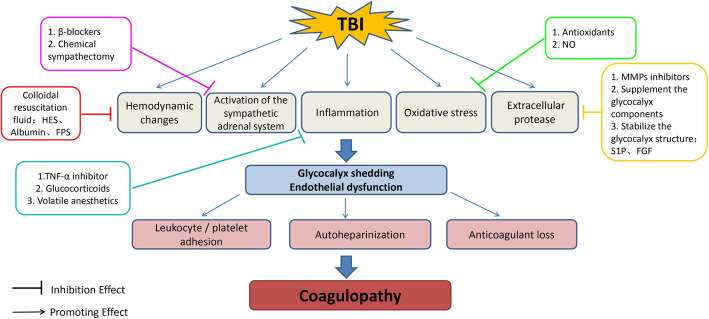
Summary of the main mechanisms of EG injury induced TBI coagulopathy and the potential treatment for protecting glycocalyx. After TBI, there are many pathological changes can induce glycocalyx damage and the imbalance between coagulation and anticoagulation, thereby causing coagulopathy. Effects of different treatment measures (various colors) are pointed out with a closed tip arrow, at every stage where benefits have been observed. HES, hydroxyethyl starch; FPS, fresh frozen plasma; NO, nitric oxide; S1P, sphingosine-1 phosphate; FGF, fibroblast growth factor

In the following, we will summarize potential damage mechanisms and options to restore glycocalyx function under the unique characteristics of TBI (Table 1, Fig. 2).

**Table 1:** The possible factors of EG damage in TBI coagulopathy

Factors	Types
**Natriuretic peptide**	ANP, BNP, CNP
**Catecholamine**	NE, Epi
**Inflammatory mediators**	TNF-α, IL-1β, IL-6, IL-8, IL-10
**Angiopoietin**	Ang-2
**Chemotactic factors**	fMLP
**Reactive species**	ROS, RNS
**Histone deacetylase**	HDAC
**Extracellular proteases**	MMP-1, MMP-2, MMP-7, MMP-9, MMP-14, MMP-16, ADAM-10, ADAM-15, ADAM-17 ADAMTS-1, ADAMTS-4 Heparanase, hyaluronidase, thrombin, plasmin, elastase, lysosomal cathepsin B, tryptase β

*EG* endothelial glycocalyx, *TBI* traumatic brain injury, *ANP* atrial natriuretic peptide, *BNP* brain natriuretic peptide, *NE* norepinephrine, *Epi* epinephrine, *TNF* tumor necrosis factor, *IL* interleukin, *Ang* angiopoietin, *fMLP* N-formylmethionyl-leucyl-phenylalanine, *ROS* reactive oxygen species, *RNS* reactive nitrogen species, *HDAC* histone deacetylase, *MMP* matrix metalloproteinase, *ADAM* a disintegrins and metalloproteinase, *ADAMT* a disintegrin and metalloproteinase with thrombospondin motif

### Hemodynamic changes

Under physiological conditions, the vascular EG, as a mechanical sensor of blood flow, can reduce shear forces on cellular surfaces to a negligible level [70]. However, under pathological conditions, the shear force acting on the surface of endothelial cells can affect the structure of the glycocalyx by (i) changing its molecular composition, (ii) affecting the biosynthesis of new components, or (iii) activating proteases and lyases synthesized by endothelial cells [71–73]. Trauma patients often suffer from extensive tissue damage, hemorrhagic shock, and insufficient tissue perfusion, resulting in hemodynamic change. It has been shown that the decrease in plasma colloidal osmolality is related to the magnitude of the injury and the increase in syndecan-1 shedding, which leads to an increase in the demand for blood transfusion and resuscitation [74]. Fluid resuscitation is often used in critically ill patients to increase cardiac output and reverse pathological hypoperfusion, thereby preventing organ damage. Experimental and clinical evidence, however, suggests that fluid resuscitation only exerts short-term hemodynamic improvements, but may lead to long-term abnormal cardiovascular function, microcirculatory dysfunction, and increased demand for vasopressor therapy, with all significantly related mortality [75, 76]. These derangements are mainly due to increased release of atrial natriuretic peptide (ANP) following mechanical pressure caused by high blood volume, which mediates glycocalyx shedding increased vascular permeabilit y[77]. In addition to ANP, B- (BNP) and C-type (CNP) natriuretic peptide can also induce damage to the glycocalyx, which may explain the long-term adverse outcomes in clinical studies when recombinant human BNP (nesiritide) or ANP (carperitide) have been administered to improve cardiac function or reduce myocardial infarction injur y[78]. However, there is no proven mechanism yet described by which natriuretic peptides cause glycocalyx shedding. The concentration-dependent glycocalyx shedding provided by ANP in the experimental setting suggests an activation of natriuretic peptide receptors (NPRs).

Hemodynamic changes after TBI frequently show a close relation to the extent and magnitude of brain injury [79]. In fact, the change in systemic blood volume remains the most common factor for secondary injury in TBI; this includes the reduction in absolute blood volume, for example in hemorrhagic shock, as well as the reduction in relative blood volume, for example due to peripheral vasodilation and reductions in effective blood volume, which may cause mean arterial pressure to decline [80, 81]. Among patients with severe TBI, about one in three patients had hypotension and hypoxemia prior to rescue and resuscitation. Even on the intensive care unit (ICU), more than 25% of patients with severe TBI suffer from ongoing or periodic hypotension [82]. Besides, TBI can indirectly induce hypotension and subsequent coronary hypoperfusion through sympathetic overactivation and stress-induced cardiac dysfunction, thereby exacerbating systemic hemodynamic changes. In experimental combined TBI and hemorrhagic shock models, shedding of glycocalyx was determined and closely related to the occurrence of hemocoagulative dysfunction including coagulopathy [48]. Unfortunately, the correlation between hemodynamic changes in TBI and damage to the glycocalyx was not analyzed in further detail.

#### Potential therapeutic options

Given that changes in blood volume can be associated with increased glycocalyx degradation, protecting and restoring the vascular EG may be an important goal for clinical management. Therefore, the type of fluid resuscitation and the appropriate fluid volume are of yet to be specified significance to maintain the glycocalyx integrity. Currently, colloidal resuscitation is recommended to protect the integrity of the glycocalyx. Margraf and co-workers have reported that 6% hydroxyethyl starch (HES 130/0.4) diminishes glycocalyx degradation and decreases vascular permeability during systemic and pulmonary inflammation in mice as compared to resuscitation with crystalloids [83]. Sphingosine-1 phosphate (S1P) is a sphingolipid in plasma that plays a critical role in the cardiovascular and immune systems. Serum albumin and high-density lipoprotein (HDL) carry ~ 90% of the S1P, and both elicit the release of S1P from RBCs. The S1P receptor most abundantly expressed on ECs is S1P1, and S1P can protect the glycocalyx via the S1P1 receptor. Albumin, a colloid commonly used for volume resuscitation, has been considered to have protective effects on the glycocalyx because it may carry S1P derived from red blood cells into endothelial cells and mediate glycocalyx recovery by inhibiting matrix metalloproteinases (MMPs) activity [84, 85]. A series of other preclinical studies have found that, compared with the control group and lactated Ringer’s solution (LRS) resuscitation group, fresh frozen plasma (FFP) may exert protective effect on glycocalyx degradation in experimental animals when exposed to hemorrhagic shock [86, 87]. Therefore, a more in-depth understanding of the vascular EG may help to refine fluid resuscitation programs and strategies for critically ill patients with TBI. It also has been suggested that B-type receptor inhibitors, for example anantin, or inhibitors of natriuretic peptide receptor signal transduction may have a protective effect on glycocalyx. No such drugs have yet been approved for human use so far.

### Activation of the sympathetic adrenal system

Preclinical and clinical studies have shown that catecholamine neurotransmitters are in disorder in the presence of central degenerative diseases and brain injury. TBI often leads to excessive activation of the sympathetic nervous system, which is manifested by the increased activity of catecholamine synthetases such as tyrosine hydroxylase and the surge of catecholamine transmitters (e.g., dopamine, norepinephrine and epinephrine) not only aggravates brain tissue damage but also relates closely to the pathological events that lead to secondary brain and systemic injury [88–90]. At present, a large body of evidence has shown that the sympathetic adrenal system is triggered in trauma including TBI, septic shock, ischemia-reperfusion and acute myocardial infarction, leading to endothelial cell injury, glycocalyx shedding, and abnormal blood coagulation [8, 89]. This may be interpreted as an adaptive response at first, which is conductive to the transformation from a hypercoagulable to a hypocoagulable state in order to maintain microvessels open and to promote blood perfusion and oxygen delivery within various tissues/organs [90]. In addition to effects on the systemic circulatory system, sustained high catecholamine levels can also directly damage endothelial cells, leading also to shedding of the glycocalyx including its components. Martin and co-workers [91] have directly used different titrations of norepinephrine (NE) and epinephrine (Epi) to stimulate HUVECs under simulated conditions of shock and found that catecholamines may cause glycocalyx damage and endothelial activation under stress conditions in a dose-dependent manner. However, the current research on correlation of catecholamines and glycocalyx shedding is limited to descriptive and analyses only with specific damage. Mechanisms still need to be further explored.

#### Potential therapeutic options

Many experimental studies have found that treatment with nonselective β-blockers (BBs) (e.g., propranolol and clonidine) can reduce cerebral edema, blood-brain barrier permeability, and glycocalyx damage at 24 h in animals exposed to TBI [92]. Clinical studies also confirmed that early use of β-blockers after TBI could (i) protect end organs that are vulnerable to catecholamine damage, (ii) improve microcirculation, and (iii) increase survival [93, 94]. Similarly, chemical sympathectomy has also been shown to reduce inflammation, glucocorticoid shedding, and disturbed hemostasis in experimental animals with acute traumatic coagulopathy [95]. However, clinical guideline has yet been established for the use of BBs in TBI patients.

### Inflammation

In most tissues, including the central nervous system, inflammatory responses are elicited after both trauma and ischemia [96]. Glycocalyx acts as the first barrier to endothelial injury; studies have demonstrated the correlation between inflammatory mediators (such as TNF-α, IL-1β, IL-6, and IL-10) and glycocalyx shedding [97]. Many clinical syndromes of systemic inflammation, including sepsis, major surgery, trauma, atherosclerosis, and persistent hyperglycemia, etc., can cause diffuse and persistent damage to the glycocalyx [31]. The combination of TBI and shock results in an immediate activation of both coagulation and complement systems with subsequent EG shedding, protein C activation, and inflammation [48]. Many studies have shown that adherent leukocytes can directly damage the endothelium; however, in the absence of leukocyte-endothelial cell adhesion, Henry and co-workers have treated endothelial cells with TNF-α for 20 min and observed glycocalyx shedding [98]. It may be that TNF-α increases endothelial-derived free radicals or activates surface-bound proteases and phospholipases, thereby causing glycocalyx damage. Angiopoietin 2 (Ang-2) is a protein secreted by endothelial cells under inflammatory stimulation which can inhibit the anti-inflammatory signal induced by Ang-1 activation of the tyrosine kinase-2 (Tie2) receptor which is also considered a key mediator of glycocalyx degradation. Lukasz and co-workers [99] have used Ang-2 to treat HUVECs and mice, which can cause rapid degradation of the EG both in vivo and in vitro, indicating that inhibition of Ang-2 and activation of Tie2 may be potential therapeutic targets for glycocalyx protection. In addition, n-formyl-met-leu-phe (fMLP), a potent neutrophil chemotactic peptide, can also cause glycocalyx shedding and intercellular adhesion molecule 1 (ICAM-1) exposure by binding to specific G protein-coupled receptors on endothelial cells, thus promoting leukocyte adhesion in rat mesenteric capillaries [100]. During inflammation, the signal of glycocalyx repair mediated by FGF is significantly suppressed [44]. Therefore, enhancing the glycocalyx repair signal may be a potential treatment method to restore the glycocalyx layer and improve its function.

#### Potential therapeutic options

Data from experimental animal and cell experiments indicate that glycocalyx injury occurs within hours after inflammation, but it takes 5–7 days to return to normal. Therefore, inhibition of inflammatory response may represent a promising target for glycocalyx protection and restoration. TNF-α is one of the critical mediators in both acute and chronic inflammation. The clinical use of TNF-α inhibitor, Etanercept, an analog of TNF-α receptor, can significantly reduce endotoxin-induced glycocalyx component shedding, coagulation activation, and vascular dysfunction [101]. Since the vascular permeability of macromolecules is significantly increased during inflammation, glucocorticoids are usually used to prevent edema and swelling between tissues. Antithrombin is an important inhibitor of the coagulation system and has specific anti-inflammatory effects on endothelial cells. In an isolated heart model, Chappell and co-workers found that hydrocortisone and antithrombin can significantly reduce glycocalyx shedding after ischemia/reperfusion and TNF-α-induced inflammation [102, 103]. Glucocorticoids can also stabilize mast cells and prevent degranulation, thereby reducing inflammation on the glycocalyx. Although glucocorticoids have positive effects, the exact mechanism through which they act on EG is still unclear. Other volatile anesthetics, such as sevoflurane and isoflurane [104, 105], have also been found to have anti-inflammatory effects, which may potentially attenuated EG destruction caused by inflammation. These suggest that sedative and analgesic drugs with anti-inflammatory effects used in the clinical management of TBI may be beneficial to protect glycocalyx and improve microcirculation.

### Oxidative stress

Oxidative stress refers to the process of tissue damage caused by an imbalance between the oxidation and the antioxidant system when the body is subjected to various harmful stimuli [106]. As early as 1986, Kontos and co-workers have shown that a large number of free radicals are generated after TBI [106, 107]. Free radicals are by-products of the body metabolism, which are promptly eliminated by the antioxidant mechanism under physiologic conditions. After TBI, due to the decrease in local cerebral blood flow in affected areas, the oxidation level within brain tissue cells is enhanced, and the antioxidant capacity and the ability to scavenge free radicals are weakened, automatically leading to the accumulation of free radicals. Severe TBI patients with shock can also experience systemic ischemia and tissue hypoxia, which will eventually cause a vicious circle.

Oxidative stress can occur in cardiopulmonary surgery [73], severe trauma [108], massive blood loss [31], hypoxia [109], and ischemia-reperfusion [110]. In all cases, the EG covering the microvasculature is attacked and damaged by reactive oxygen and nitrogen species, ROS and RNS respectively, which can cause EG injury and increase in plasma syndecan-1 and HS levels [110]. At present, the detrimental effects of oxidative stress on glycocalyx are mostly derived from various experimental models and clinical studies of ischemia/reperfusion and hypoxia [111]. In vivo studies have shown that the EG shed rapidly after ischemia-reperfusion [112, 113], and the injury of the glycocalyx was also confirmed in patients who experienced local ischemia during clinical vascular surgery [73]. The mechanisms behind these observations are still unclear. Potential mediators may include excessive free radicals, complement activation, TNF-α expression, mast cell degranulation, and protease (especially metalloproteinase) activation. Ali and co-workers have reported that MMP is the primary cellular protease involved in the degradation of EG induced by oxidative stress in vitro [114]. After oxidative stress, histone deacetylase (HDAC) mediates the upregulation of MMPs and the downregulation of tissue inhibitors of metalloproteinases (TIMPs), leading to the shedding of GAGs and extracellular superoxide dismutase (SOD) and aggravating oxidative stress injury [115].

#### Potential therapeutic options

Several studies have shown that antioxidants can protect tissues and organs from oxidative stress damage [116, 117]. It may be speculated this holds also true in the context of glycocalyx damage. As an important signal molecule of vascular cells, low-dose NO can prevent oxidative damage. Bruegger and co-workers [112] found that the use of NO prior to myocardial reperfusion can reduce coronary artery leakage and edema formation, and has a glycocalyx protective effect. The premise of this protective effect is that the glycocalyx is not degraded in advance.

### Extracellular protease

The endothelial glycocalyx, a poly glycoprotein complex covering the surface of the endothelial cell, is a component to the extracellular matrix (ECM) and is in direct contact to various components within the blood stream, including a range of proteases. A large number of studies have confirmed the effect of various “sheddases” on the glycocalyx in different pathological settings, and different “sheddases” may have selective targeting effects on various components of the glycocalyx (Table 2) [64, 114, 118]. The most common is the superfamily of MMPs, with more than 20 members, including “classical” MMPs, membrane-bound MMPs (MT-MMPs), disintegrins and metalloproteinases (ADAMs), and disintegrin and metalloproteinase with thrombospondin motifs (ADAMTSs) [119]. One of the most in-depth studies of these enzymes is their ability to catalyze the cleavage of the extracellular domain of transmembrane proteins and reduce their cell surface levels [120–122]. The hypothesis that MMPs can cleave the EG and promote the shedding of different components under pathological conditions has been fully supported [123–126]. MMPs were stored in the endothelial vesicles in an inactive form and could be released rapidly by activated endothelial cells under appropriate stimulation. The activity of MMPs is regulated by a group of endogenous proteins, called TIMPs, which bind to the active sites of activated MMPs to prevent their activation. Besides MMPs, heparanase [127] and hyaluronidase [53] can specifically cleave HS and HA components in glycocalyx. According to limited reports, thrombin [128], plasmin [129], elastase [130], tryptase [131], and cathepsin B [132], which can induce glycocalyx shedding, are also potential “sheddases.”

**Table 2:** Shaddases of EG and the studies in TBI

Candidate sheddases	EG components	Studies in TBI
**MMPs**		
MMP-1	Syndecan-1,HS	Neuroinflammation
MMP-2	Syndecan-1,-2,CS	BBB dysfunction, neuroinflammation, apoptosis
MMP-7	Syndecan-1,-2,CS	Neuroinflammation
MMP-9	Syndecan-1,-2,CS	BBB dysfunction, neuroinflammation, apoptosis
MMP-14(MT1-MMP)	Syndecan-1	
MMP-16(MT3-MMP)	Syndecan-1	
**ADAMs**		
ADAM-10	EMCN	Poor synaptic recovery
ADAM-15	CD44	
ADAM-17	Syndecan-1,-4, EMCN	Neuroinflammation, neural progenitor cell migration, neurogenesis
**ADAMTSs**		
ADAMTS-1	Syndecan-4	Neuroinflammation
ADAMTS-4	Syndecan-4	Neuroinflammation
**Heparanase**	HS	
**Hyaluronidase**	HA	BBB dysfunction, behavioral decrements, neuroinflammation
**Thrombin**	Syndecan-1,-4	BBB dysfunction, apoptosis, cerebral ischemia, excitotoxicity
**Plasmin**	Syndecan-4	Cerebral hemorrhage, inflammation, coagulopathy
**Elastase**	Syndecan-1,-4	Neuroinflammation, oxidative stress, apoptosis
**Lysosomal cathepsin B**	Syndecan-1, HS	Neuroinflammation, apoptosis, neurodegeneration
**Tryptase β**	Syndecan-1, HS	Neuroinflammation, apoptosis

*EG* endothelial glycocalyx, *TBI* traumatic brain injury, *MMPs* matrix metalloproteinases, *MT-MMP* membrane-bound MMP, *ADAMs* a disintegrins and metalloproteinases, *ADAMTS* a disintegrin and metalloproteinase with thrombospondin motifs, *HS* heparin sulfate, *HA* hyaluronic acid, *CS* chondroitin sulfate, *EMCN* endomucin, *CTF* C-terminal fragments

At present, many proteases have been reported to play important roles in various nervous system diseases and injuries, with their expression up-regulated and enzyme activity increased after TBI [133]. These proteases are responsible for the cleavage of transmembrane proteins in neurons, glial cells, and endothelial cells, resulting in the release of the extracellular domain, which mediates neural function injury as well as repair after TBI. In the adult central nervous system (CNS), the expression level of most MMPs is low or uncertain [134–138], only 17 out of 30 ADAMs [133, 139–141] play a role in the brain, and the literature on the expression of ADAMs in CNS pathology is very limited. Most of the “sheddases” of the glycocalyx display expression and activity changes in the context of TBI [53, 130, 142–152], but research has yet mainly focused on inflammatory response and neural repair processes (Table 2). The relationship between these “sheddases” of glycocalyx injury and secondary coagulopathy after TBI needs further study.

#### Potential therapeutic options

Dane and co-workers [153] have reported that the glomerular EG damage induced by intravenous injection of hyaluronidase takes 4 weeks to return to physiologic levels. Therefore, it is important to take measures to prevent or reduce glycocalyx damage of vascular endothelial cells early when injury factors are acting.

Specific inhibitors of “sheddases” can be selected to prevent degradation of the glycocalyx/endothelial surface layer. Although many studies have shown that nonselective inhibitors of metalloproteinases such as phenanthroline, doxycycline can reduce damage promoted by inflammation and oxidative stress on the glycocalyx [154, 155]; early administration of tranexamic acid (TXA) can inhibit the activities of ADAM-17, TNF-α, and MMP-9 and may protect the glycocalyx-vascular barrier, reduce the occurrence of post-traumatic bleeding, and improve survival [156]. However, the currently available MMP inhibitors are not selective for specific protease subtypes and do not have the same inhibitory activity against other members of the family. In addition, combined with the similar structure of MMP members and the complexity of the activation pathway, the continued inhibitory effect of MMPs must be considered. In addition, in combination with the similarity of the structure of MMPs and the complexity of the activation pathway, the duration of MMP inhibition and the later role of these proteases in CNS repair after brain injury must be considered. All these aspects have led to a very limited application and efficacy of MMP inhibitors in clinical research so far. In addition, Grundman and co-workers [157] have found that antithrombin III can bind tightly to the glycocalyx, by promoting the release of prostacyclin, inhibiting other “sheddases” activation pathways, and reducing glycocalyx damage.

The specific supplementation of glycocalyx components may represent another therapeutic avenue. For example, Broekhuizen and co-workers have reported that after 8 weeks of treatment with sulodexide, a mixture of glycocalyx GAG precursors, glycocalyx damage to the sublingual and retinal vascular beds was reduced, and capillary albumin leakage trended towards normal in diabetic patients [158]. Hayashida and co-workers have found that intraperitoneal injection of HS can reduce the level of inflammatory proteins underlying glycocalyx damage in liver and lung tissues of mice with endotoxemia [159]. Supplementation of heparin analogs can also reduce the plasma levels of syndecan-1 and thus alleviating glycocalyx injury [160].

A final therapeutic concept that needs further exploration is the stabilization of the glycocalyx structure. S1P is a sphingolipid synthesized by red blood cells, which can regulate the synthesis of glycocalyx components by binding to receptors on the surface of endothelial cells. Adamson and co-workers [85] have found that a certain amount of S1P (100–300 nmol/L) may promote and maintain EG stability. In addition, HS fragments produced by glycocalyx shedding in the circulation can activate FGF, which binds to FGF receptors to activate glycocalyx repair molecules by initiating signal transduction pathways, such as exostosin-1 (a kind of the enzyme responsible for the synthesis of heparan sulfate), and mediates the physiological repair of the glycocalyx.

## Conclusion

There is increasing interest and awareness for the potential role of vascular EG damage in the pathophysiological sequelae after TBI. Selecting the endothelial glycocalyx as a therapeutic target to prevent and limit glycocalyx damage and to protect the vascular endothelial barrier function provides a new concept for both TBI and coagulation management. The specific mechanisms by which damage to the glycocalyx is promoted are still unknown and need further exploration. The majority of studies yet conducted in the field have been experimental animal studies whose results clearly need verification in the clinical arena.

## Data Availability

Not applicable.

## Abbreviations

TBI: Traumatic brain injury
DAMPs: Damage-associated molecular patterns
EG: Endothelial glycocalyx
GAGs: Glycosaminoglycans
PGs: Proteoglycans
HA: Hyaluronic acid
HS: Heparin sulfate
CS: Chondroitin sulfate
HSPG: Heparan sulfate proteoglycan
EC: Endothelial cells
HES: Hydroxyethyl starch
NO: Nitric oxide
HUVECs: Human umbilical vein endothelial cells
RBCs: Red blood cells
ANP: Atrial natriuretic peptide
BNP: B-type natriuretic peptide
CNP: C-type natriuretic peptide
S1P: Sphingosine-1 phosphate
MMPs: Matrix metalloproteinases
BBs: β-blockers
Ang-2: Angiopoietin 2
Tie2: Tyrosine kinase-2
FGF: Fibroblast growth factor
TIMPs: Tissue inhibitors of metalloproteinases
MT-MMPs: Membrane-bound MMPs
ADAMs: A disintegrins and metalloproteinases
ADAMTSs: A disintegrin and metalloproteinase with thrombospondin motifs
CNS: Central nervous system
